# The Dose-Dependent Pleiotropic Effects of the UBB^+1^ Ubiquitin Mutant

**DOI:** 10.3389/fmolb.2021.650730

**Published:** 2021-03-26

**Authors:** Katarzyna Banasiak, Natalia A. Szulc, Wojciech Pokrzywa

**Affiliations:** Laboratory of Protein Metabolism, International Institute of Molecular and Cell Biology in Warsaw, Warsaw, Poland

**Keywords:** molecular misreading, UBB^+1^, ubiquitin proteasomal system, neurodegeneration, proteotoxic stress response, ROS generation and cytotoxicity, cellular viability

## Abstract

The proteolytic machinery activity diminishes with age, leading to abnormal accumulation of aberrant proteins; furthermore, a decline in protein degradation capacity is associated with multiple age-related proteinopathies. Cellular proteostasis can be maintained *via* the removal of ubiquitin (Ub)-tagged damaged and redundant proteins by the ubiquitin-proteasome system (UPS). However, during aging, central nervous system (CNS) cells begin to express a frameshift-mutated Ub, UBB^+1^. Its accumulation is a neuropathological hallmark of tauopathy, including Alzheimer’s disease and polyglutamine diseases. Mechanistically, in cell-free and cell-based systems, an increase in the UBB^+1^ concentration disrupts proteasome processivity, leading to increased aggregation of toxic proteins. On the other hand, a low level of UBB^+1^ improves stress resistance and extends lifespan. Here we summarize recent findings regarding the impact of UBB^+1^ on Ub signaling and neurodegeneration. We also review the molecular basis of how UBB^+1^ affects UPS components as well as its dose-dependent switch between cytoprotective and cytotoxic roles.

## Introduction

Age-related impairment of protein degradation affects protein homeostasis (proteostasis) networks, causing enhanced accumulation of damaged proteins that can be cytotoxic and shorten lifespan. The primary proteolytic component of the cellular proteostasis network is the ubiquitin-proteasome system (UPS), which initiates turnover of unwanted substrates *via* covalent attachment of the evolutionarily conserved protein ubiquitin (Ub) (Buchberger et al., 2010; Popovic et al., 2014; Balchin et al., 2016). More than 2 decades ago, van Leeuwen and colleagues, while studying Alzheimer’s plaques in postmortem brains, identified a frameshift-mutated form of Ub currently known as UBB^+1^ (van Leeuwen et al., 1998). Subsequent studies confirmed the involvement of UBB^+1^ in several other neurodegenerative diseases (i.e., Pick disease and progressive supranuclear palsy) as well as in polyglutamine (polyQ) diseases (i.e., Huntington’s disease and spinocerebellar ataxia type 3). Furthermore, UBB^+1^ accumulation has been linked to disease onset and progression (Fergusson et al., 2000; Fischer et al., 2003; de Pril et al., 2004; Hol et al., 2005; Yim et al., 2014). Nonetheless, UBB^+1^ has also been found in healthy neurons and other cell types, including monocytes and hepatocytes (French et al., 2001; Fischer et al., 2003; Fratta et al., 2004). Recent findings highlight the positive effects associated with UBB^+1^ expression. For example, UBB^+1^ synthesis reduces amyloid-β- (Aβ-) related toxicity (Verheijen et al., 2018), and in yeast, a low level of UBB^+1^ expression prevents reactive oxygen species (ROS) accumulation and limits apoptosis, consequently increasing cellular life span (Muñoz-Arellano et al., 2018). Here, we discuss at least some of the many faces of UBB^+1^ in proteostasis maintenance.

## Molecular Misreading Leads to UBB^+1^ Expression

**FIGURE 1:**
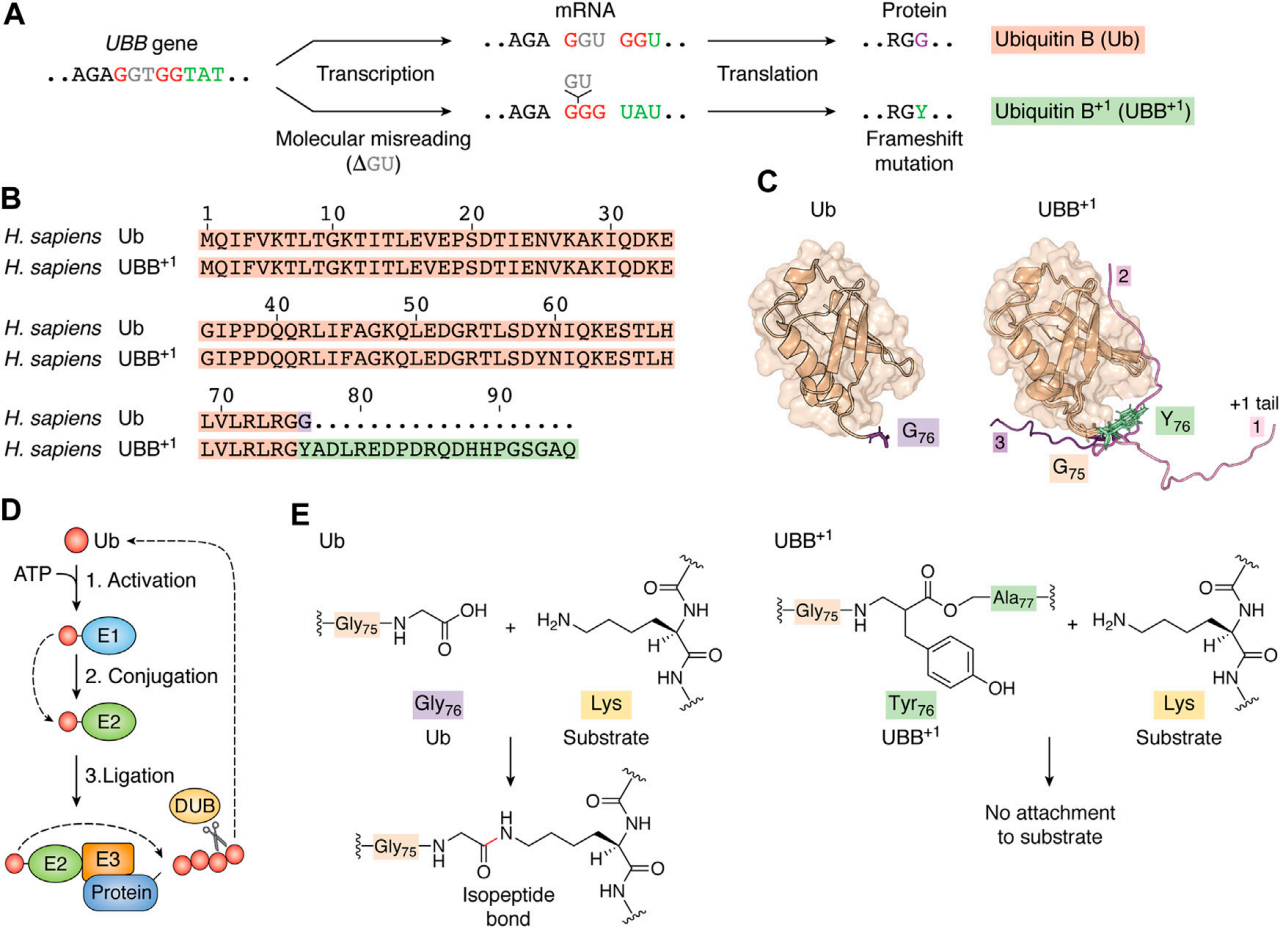
Transcription error leads to the expression of a mutated form of ubiquitin. **(A)** Molecular misreading of Ub mRNA sequences results in “GU” nucleotide deletion, which introduces a frameshift mutation that results in UBB^+1^ expression. **(B)** UBB^+1^ differs from wild-type Ub by the G76Y missense mutation and a 19-amino acid C-terminal extension. **(C)** The tertiary structures of both Ub (PDB ID code: 1d3z, Cornilescu et al., 1998) and UBB^+1^ (model built upon PDB structures with codes 1d3z and 2kx0) exhibit a compact and globular “β-grasp” fold that forms a hydrophobic core between an α-helix and five β-sheets. In our model of UBB^+1^, we show three distinct conformations of the extended amino acid chain. Additional 19-amino acid chains (from models 1, 2, 9 of 2kx0) were added to 1d3z structure with G76Y mutation using YASARA View (Krieger and Vriend, 2014). Each model was protonated using H++ web server version 3.2 (Gordon et al., 2005; Myers et al., 2006; Anandakrishnan et al., 2012) and optimized by running 1,500 steps of energy minimization. All calculations were performed using the AmberTools20 package (Izadi et al., 2014; Li et al., 2015; Case et al., 2020; Tian et al., 2020); optimized models were superposed and visualized in PyMOL (The PyMOL Molecular Graphics System, Version 2.2.3. Schrödinger, LLC). **(D)** At the start of the ubiquitylation cascade, a Ub-activating enzyme (E1) hydrolyzes ATP and forms a high-energy thioester bond between an internal cysteine residue and the C-terminus of Ub. Activated Ub is then passed on to Ub-conjugating enzymes (E2s), which form similar thioester-linked complexes with Ub. Finally, Ub is covalently attached to lysine sidechains of the substrate protein or another Ub (generating polyUb chains) with assistance from ubiquitin-protein ligases (E3s). Deubiquitylation enzymes (DUBs) modulate the length and topology of polyUb and recycle Ub. Finally, the proteasome complex recognizes ubiquitylated proteins and degrades them into short peptides *via* proteolysis. **(E)** Ub is attached to its substrate *via* an isopeptide bond between the C-terminal glycine residue of Ub and a lysine residue in the substrate. The G76Y mutation prevents the formation of an isopeptide bond between the UBB^+1^ C-terminus and lysine residues in protein substrates.

In humans, Ub is encoded by four genes, including UB-ribosomal fusion genes, i.e., Uba52, and RPS27A, which encode a single copy of Ub fused to a ribosomal protein, and polyubiquitin genes, i.e., UBB and UBC, which consist of repeats of monoubiquitin coding units (Wiborg et al., 1985; Finley et al., 1989; Baker and Board, 1991). In addition, several Ub pseudogenes have been identified, including the recently characterized UBB pseudogene 4 (Dubois et al., 2020). Reduced Ub levels caused by UBB inactivation lead to many disorders, including adult-onset obesity and hypothalamic neurodegeneration (Ryu et al., 2008) and dysregulation of neuronal stem cell self-renewal (Ryu et al., 2014). Furthermore, abnormal transcription leads to the formation of a mutated form of ubiquitin, UBB^+1^. Molecular misreading leads to dinucleotide deletions (CU, GA, GU) in mRNA leading to a +1 reading frame shift, resulting in the synthesis of a “+1 protein” with abnormal extensions (Figure 1A) (van Leeuwen et al., 1998; van Leeuwen et al., 2000; van Leeuwen et al., 2002). Intensification of this molecular misreading and an accumulation of UBB^+1^ in multiple areas of the brain is a hallmark of neurodegeneration, including that associated with Alzheimer’s disease (van Leeuwen et al., 1998; van Leeuwen et al., 2000; van Leeuwen et al., 2002). The exact cause of these errors requires further research; however, potential mechanisms for the generation of mutant transcripts include inappropriate RNA polymerase activity at repetitive DNA sequences or ribosome-mediated frameshifting (Wills and Atkins, 2006; Verheijen et al., 2017).

## UBB^+1^ Interferes With Deubiquitylation and Proteasome-Mediated Degradation

**FIGURE 2:**
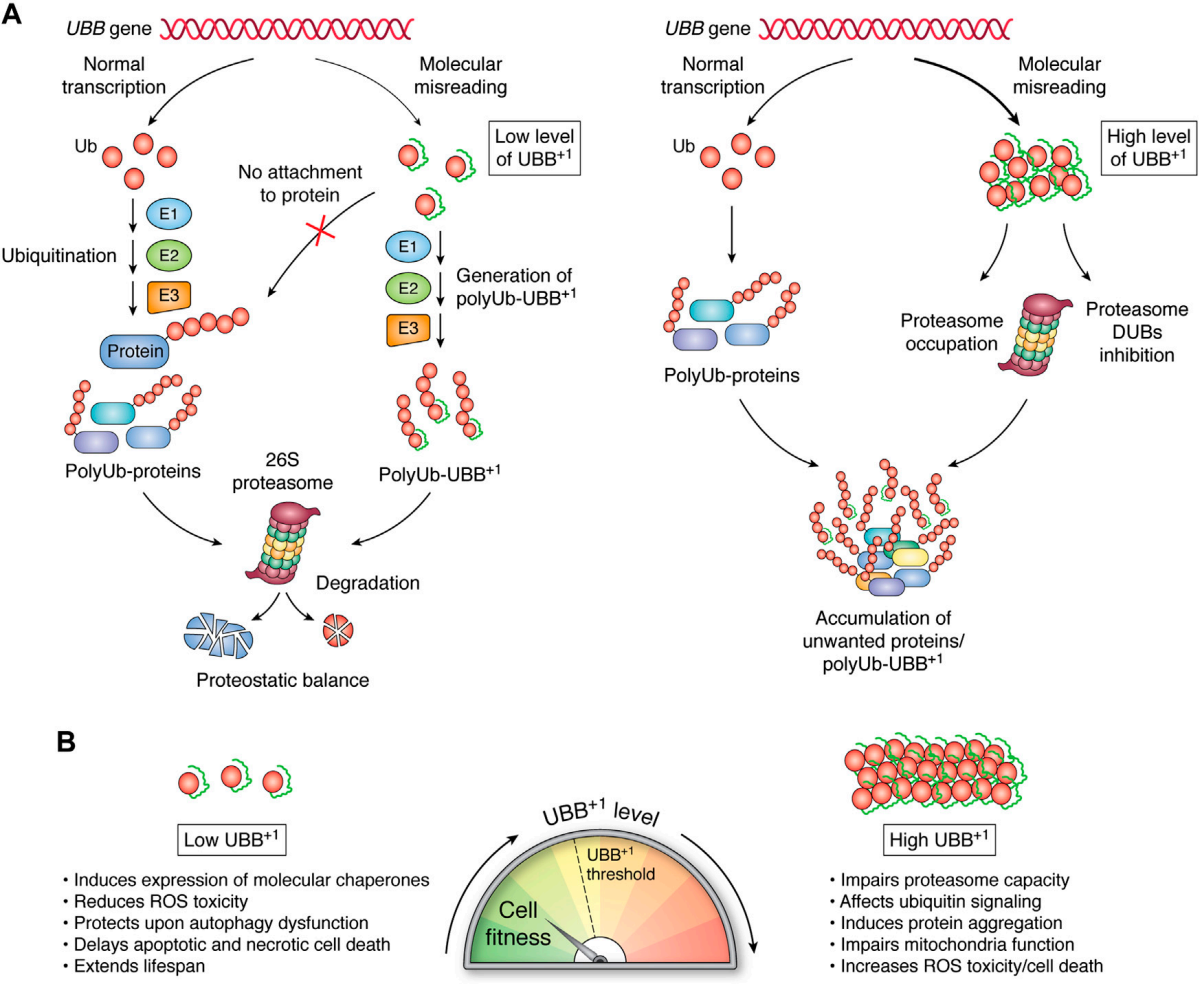
Pleiotropic effects of UBB^+1^ depend on its expression level. **(A)** E1, E2, and E3 enzymes are the sole pathway by which Ub molecules are linked to create polyUb on substrate proteins. UBB^+1^ is present in cells as a monomer that can be ubiquitylated at internal lysine residues *via* the same enzymatic cascade; however, it cannot be attached to proteins. The proteasome can efficiently recognize and proteolyze a low level of Ub-UBB^+1^ chains. Increased levels of Ub-UBB^+1^ might impair proteasomal capacity and DUB activity, leading to the accumulation of aberrant proteins designated for degradation. **(B)** The functionality of cellular processes correlates with the level of UBB^+1^ expression.

Ub attachment (ubiquitylation) is mediated by an enzymatic cascade involving Ub-activating enzymes (E1), Ub-conjugating enzymes (E2), and Ub ligases (E3). A C-terminal GG motif is necessary for Ub activation and conjugation to target proteins. PolyUb chains are assembled *via* an isopeptide linkage between the lysine residue of the previous Ub and the C-terminal glycine residue of the subsequent subunit. Deubiquitylation enzymes (DUBs) modulate the size and topology of polyUb (Figure 1D) (Komander and Rape, 2012). UBB^+1^ differs from wild-type Ub due to a G76Y mutation and a flexible 19-amino acid extension (Ko et al., 2010; Munari et al., 2018), (Figure 1B). We prepared a Ub model carrying the G76Y mutation and visualized three different conformations of the 19-amino acid extension (Figure 1C). Substitution of a glycine at residue 76 interrupts the C-terminal GG motif, which prevents its activation by E1, attachment to a substrate’s lysine, and processing by certain DUBs (Figures 1E, 2A) (Lam et al., 2000; Krutauz et al., 2014). This Ub mutant can be ubiquitylated at all lysine residues to serve as a proximal unit in polyUb chains. Studies in cells and mice have shown that UBB^+1^ is ubiquitously present in K29-, K48-, and K63-linked ubiquitin chains (Lam et al., 2000; Lindsten et al., 2002; van Tijn et al., 2012; Akutsu et al., 2016).

The conjugating enzyme UBE2K can directly interact with UBB^+1^ to initiate its ubiquitylation, which can be mediated by the E3s TRIP12 and HUWE1 (Park et al., 2009; Poulsen et al., 2009; Ko et al., 2010). In general, polyUb-UBB^+1^ are recognized by the proteasome, but they are efficiently degraded when UBB^+1^ is expressed under basal conditions (Figure 2A) (Lindsten et al., 2002; Fischer et al., 2003; van Tijn et al., 2007; van Tijn et al., 2010; Krutauz et al., 2014). Cellular UBB^+1^ accumulation promotes proteasome dysfunction, although not *via* direct inhibition of the proteosome’s hydrolytic activity. PolyUb-UBB^+1^ can be recognized by the substrate-shuttling factor for the proteasome, HHR23A and UBQLN1, and the proteasomal ubiquitin-receptor Rpn10 (Chojnacki et al., 2016; Munari et al., 2018). However, the 19-amino acid C-terminal extension of UBB^+1^ prevents the entire molecule from being effectively pulled in and reaching the 20S catalytic sites (Verhoef et al., 2008; Shabek et al., 2009). Thus, polyUb-UBB^+1^ can occupy proteasomes, thereby preventing the turnover of polyUb substrates (Figure 2A). UBB^+1^ also blocks the activity of proteasome-related DUBs, including Ubp6, which selectively cleave Lys48-linked Ub, thereby promoting substrate translocation into the 20S proteasome. Rpn11, a proteasomal subunit with DUB activity, is also inhibited by UBB^+1^ (Figure 2A) (Krutauz et al., 2014). Cytosolic DUBs also encounter obstacles when processing Ub-UBB^+1^ chains. For example, Usp5 recognizes the C-terminal glycine residue of Ub, which is absent in UBB^+1^; thus, Usp5 cannot remove UBB^+1^ subunits. YUH1/UCH-L3 DUB can hydrolyze the C-terminal extension of UBB^+1^, releasing, however, another conjugation-deficient ubiquitin Ub (G76Y) (Dennissen et al., 2011). Thus, the accumulation of degradation-resistant polyUb-UBB^+1^ and their impact on DUBs may correspond to the expansion of unwanted proteins during neurodegeneration. Moreover, Chojnacki and colleagues showed that the K63-linked Ub-UBB^+1^ chain is resistant to disassembly by the K63-specific DUB AMSH (Chojnacki et al., 2016). The effects that UBB^+1^ might have on K63-regulated processes, including DNA repair, cell signaling, and trafficking, remain to be explored.

## UBB^+1^ Expression Accompanies Neurodegeneration

Neuronal UBB^+1^ accumulation is a characteristic of neurodegenerative disorders, especially tauopathies. The link between UBB^+1^ and Alzheimer’s disease (AD) is the most extensively studied and is the subject of several reviews (van Leeuwen et al., 2006; Chadwick et al., 2012; Chen and Petranovic, 2016; Seynnaeve et al., 2018). Recently, UBB^+1^ was identified in samples from Guam island inhabitants suffering parkinsonism-dementia complex (G-PDC) (Verheijen et al., 2017). In light of current knowledge, G-PDC is the first Parkinson-related disorder with confirmed UBB^+1^ involvement. Inclusions of UBB+1 were accompanied by the UPS proteins and endoplasmic reticulum (ER) unfolded protein response (UPR^ER^) regulators, i.e., pPERK and BiP/GRP78. The UPR^ER^ is an adaptive stress response pathway that counters protein misfolding and aggregation in the ER (Hetz et al., 2020). Interestingly, depletion of the UPR^ER^ sensor Ire1 in yeast ameliorates UBB^+1^ toxicity upon oxidative stress; however, this effect is independent of the UPR^ER^ transcriptional activator Hac1 (Braun et al., 2015). Moreover, drug-induced ER stress has been found to lead to substantial UBB^+1^ accumulation in HEK293 cells (Verheijen et al., 2020). It is still unclear if and how the UPR^ER^ protects against deleterious effects of UBB^+1^ on proteostasis and neurodegeneration.

## Cytotoxicity of UBB^+1^

Braun and colleagues evaluated the cytotoxic effects of UBB^+1^ aggregation in several yeast strains with knockouts of specific UPS genes (Braun et al., 2015). They concluded that the ratio of UBB^+1^ to wild-type Ub is a more reliable indicator of UBB^+1^ cytotoxicity than proteasomal capacity itself.

### UBB^+1^ Affects ATP Synthesis, ROS Generation, and Mitochondria Organization

Recent data indicate that mitochondria are sensitive to the concentration-dependent cytotoxicity of UBB^+1^. Mitochondrial function can be evaluated by measuring cellular oxygen consumption, mitochondrial membrane potential, and ATP levels. Upon UBB^+1^ expression in yeast, the first two parameters increased after 2–3 days, while the cellular ATP level dropped (Braun et al., 2015). These observations indicate that even enhanced mitochondrial function is insufficient to prevent a metabolic crisis caused by UBB^+1^ accumulation. The cytochrome *bc*1 complex is a key component of the mitochondrial respiratory chain. Its alteration leads to abnormal ROS production and mitochondrial dysfunction (Crofts, 2004; Sousa et al., 2018). UBB^+1^ expression correlates with a significant reduction in the abundance of key components of the *bc*1 complex, i.e., Rip1 iron-sulfur protein and cytochrome c, which likely increases cellular ROS levels and decreases ATP production. Moreover, when accompanied by prolonged UBB^+1^ expression, acetate and hydrogen peroxide (H_2_O_2_) further increased the level of oxidative stress (Braun et al., 2015). Mitochondrial dynamics can also be assessed based on the expression levels of proteins related to mitochondrial fission or fusion. UBB^+1^ induction in primary human astrocytes and human astrocytoma cells has been shown to result in decreased expression of several fission-related proteins (Drp1, Fis1, and OPA3) while the concentrations of mitochondrial fusion proteins (Mfn1, Mfn2, and OPA1) were unchanged (Yim et al., 2014). In cells overexpressing UBB^+1^, mitochondria are elongated, and this phenotype has also been observed in cells treated with UPS inhibitors, which induce a significant drop in fission protein expression. Consistently, H_2_O_2_ treatment has been shown to induce astrocyte death upon UBB^+1^ accumulation (Yim et al., 2014). Dysfunction of the mitochondrial electron transport chain and mitochondrial network activates the mitochondrial (mt) unfolded protein response (UPR^mt^), a signaling cascade that aims to maintain mitochondrial protein homeostasis (Fiorese and Haynes, 2017; Shpilka and Haynes, 2018). Thus, it will be interesting to delineate the link between UBB^+1^ toxicity and the UPR^mt^ regulation.

### UBB^+1^ Disrupts Amino Acid Biosynthesis and Induces Cell Death

In UBB^+1^-overexpressing yeast cells, sixteen proteins of the mitochondrial proteome were found to have altered expression levels (e.g., Put1, Arg5, 6, Arg8, Lys1, Gpd1, and Str3). Among them, ten were previously associated with UBB^+1^-related pathologies (Braun et al., 2015). Deletion of *ARG5,6, ARG8*, and *LYS1*, which are involved in amino acid metabolism, rescued the clonogenic potential of cells expressing UBB^+1^ in response to acetate-induced oxidative stress. These findings indicate that high UBB^+1^ levels enhance the biosynthesis of the basic amino acids arginine, ornithine, and lysine, potentially aggravating its cytotoxicity. Therefore, Braun et al. (2015) decided to examine ROS production upon UBB^+1^ expression in yeast strains lacking arginine and ornithine synthesis enzymes. In the absence of upstream enzymes in the cytosolic ornithine synthesis pathway (Arg2, Arg5,6, Arg7, and Ort1), UBB^+1^ toxicity decreased under both normal conditions and stress conditions induced by acetate treatment. By contrast, the absence of enzymes downstream of cytosolic ornithine synthesis (Arg3, Arg1, and Arg4) did not affect UBB^+1^-triggered toxicity. In these phenotypes, perturbation of lysine levels appears to be irrelevant. Consequently, UBB^+1^ accumulation can influence mitochondria-associated overproduction of ornithine and arginine, key signaling molecules in the execution of cell death pathways (Braun et al., 2015). In aged human brains and brains from AD patients, the arginine and ornithine levels were altered (Rushaidhi et al., 2012; Inoue et al., 2013; Liu et al., 2014; Braun et al., 2015). However, the relationship between UBB^+1^ expression and amino acid metabolism-dependent cell death in aging requires confirmation.

### UBB^+1^ Impairs Retrograde Axonal Transport

In neurons, the microtubule cytoskeletal system is responsible for bidirectional mitochondria transport between the soma and synaptic terminals. While KIF1B kinesin plays a crucial role in anterograde transport, the dynein intermediate chain P74 is responsible for retrograde transport (Perlson et al., 2010; Maday et al., 2014). In intracellular organelles, impairment of this process leads to clogging by mitochondria in neuritic beads. In primary cortical neurons transfected with UBB^+1^, mitochondria-associated P74 levels decreased accompanied by a corresponding increase in cytosolic dynein, while KIF1B levels were unaltered (Tan et al., 2007). Consequently, UBB^+1^ expression can impair the interaction between mitochondria and dynein, thus leading to cargo detachment during retrograde axonal transport. Since impaired axonal mitochondrial transport promotes tau phosphorylation, which leads to its aggregation (Guo et al., 2020), it will be interesting to verify the impact of UBB^+1^ on this process.

## Cytoprotective Roles of UBB^+1^

### UBB^+1^ Triggers Chaperone Protein Expression

Heat shock proteins (HSPs) function as chaperones that bind misfolded polypeptides and support the refolding and recovery of native protein conformations (Lindquist and Craig, 1988; Höhfeld and Hartl, 1994; Bukau et al., 2006; Morimoto, 2008). In human neuroblastoma cells, the transcript levels of several heat shock proteins (Hsp10, Hsp40, Hsp60, Hsp70, and Hsp90a) have been found to be elevated upon UBB^**+**1^ expression (Hope et al., 2003). However, this effect is only robustly reflected at the protein level for Hsp40 and Hsp70 (only a moderate increase in Hsp90a has been found). To examine how these cells manage oxidative stress upon UBB^+1^ expression, cell survival was measured based on mitochondrial activity after tert-butyl hydroperoxide (tBHP, a strong oxidant) treatment for 24 h. Elevated production of UBB^+1^, but not Ub, mitigated tBHP cytotoxicity. Similar effects were observed for 15 μM MG132 (a reversible proteasome inhibitor) and 0.1 μM lactacystin (an irreversible proteasome inhibitor) (Hope et al., 2003). These findings suggest that UBB^+1^ is involved in the maintenance of oxidative stability *via* HSPs. Moreover, the gene encoding 14-3-3 zeta (ζ), a protein with chaperone-like activity, was highly expressed in UBB^+1^-overexpressing mice (Irmler et al., 2012; Sluchanko et al., 2012). Due to the role of 14-3-3ζ in the regulation of the unfolded protein response in the mouse hippocampus (Brennan et al., 2013), its elevated levels associated with UBB^+1^ expression might ultimately protect these cells from ER stress.

### UBB^+1^ Induces Proteotoxic Stress Resistance and Improves Cellular Viability

Muñoz-Arellano and colleagues investigated the influence of low (low_UBB^+1^) or high (high_UBB^+1^) levels of UBB^+1^ expression on yeast fitness. The incorporation of azetidine-2-carboxylic acid (AZE), a proline analogue, into de novo synthesized polypeptides disrupts the flexibility of the polypeptide backbone, thereby inducing protein misfolding stress. After incubation with AZE for 3 days, high_UBB^+1^ cells were significantly less viable than the control cells and low_UBB^+1^ cells. Since lower ROS levels were detected in the low_UBB^+1^ cells after 9 and 14 days of culture, Annexin V staining was used to check for a corresponding decrease in apoptosis. Indeed, low_UBB^+1^ cells were less apoptotic than control cells and high_UBB^+1^ cells. Moreover, control and autophagy-deficient (*∆atg1*) cells were found to cope better with protein misfolding induced with 2 mM and 4 mM AZE when UBB^+1^ is expressed at low levels. Furthermore, when yeast cells were grown in a synthetic defined (SD) medium commonly used for chronological lifespan assays, low_UBB^+1^ cells showed the highest viability. After 14 days, the average viability of the low_UBB^+1^ cells was approximately 70%, while that of the control and high_UBB^+1^ cells fell below 10% (Muñoz-Arellano et al., 2018). Under H_2_O_2_-induced oxidative stress in stationary phase cells, both low_UBB^+1^ and high_UBB^+1^ cells had improved viability compared with controls, especially in the case of aging cells (Muñoz-Arellano et al., 2018). This seems to be evolutionarily conserved as a low level of UBB^+1^ protects astrocytic cells from oxidative stress (Yim et al., 2014). Interestingly, this effect does not require the functionality of the proteasome, as its proteolytic activity similarly decreased in the low_UBB^+1^ and high_UBB^+1^ yeast cells; however, it might be related to differences in sustained expression of certain chaperones (Muñoz-Arellano et al., 2018). Further insight is needed to understand how chaperone networks are fine-tuned to maintain the cellular proteome and support UBB^+1^ positive cells’ longevity.

### UBB^+1^ Ameliorates the Aggregation of Pathogenic Proteins

Autosomal dominant AD is linked to mutations in the genes encoding β-amyloid precursor protein (APP), presenilin-1 (PSEN1), and presenilin-2 (PSEN2), which lead to impaired γ-secretase (PSEN1/PSEN2) function and improper processing of the amyloid precursor protein (APP), ultimately resulting in the formation of toxic forms of β-amyloid (Aβ) (Jankowsky et al., 2004; Bateman et al., 2011; O’Brien and Wong, 2011). To study the effect of UBB^+1^ accumulation on the development of autosomal dominant AD, mice overexpressing UBB^+1^ specifically in the postnatal brain neurons (tg line 3413) were crossbred with an AD mouse model, APPPS1 (line 85) (Fischer et al., 2009; Verheijen et al., 2018). Interestingly, the reduced γ-secretase activity of the APPPS1 animals was partially restored in APPPS1/UBB^+1^ triple transgenic animals, thus leading to fewer Aβ plaques in the brain (Chadwick et al., 2012; Verheijen et al., 2018). To determine whether UBB^+1^ expression might have broader beneficial effects, including modulation of contextual memory that reflects AD patients’ cognitive symptoms, behavioral tests have been performed in animal models. Both APPPS1 and APPPS1/UBB^+1^ mice performed worse than wild-type and UBB^+1^ mice in nest building (which provides information on general wellbeing). In a Y-maze spontaneous alternation test, however, all of the mice demonstrated similar eagerness to explore a new environment. By contrast, all of the transgenic mice performed more poorly in a Morris water maze (MWM) compared with the control mice. In conclusion, recovery of γ-secretase function due to UBB^+1^ overexpression does not appear to improve overall brain function or wellbeing. Perhaps the enhanced Aβ processing is outweighed by the negative effects of UBB^+1^ on the UPS.

## Discussion

UBB^+1^ can elicit pleiotropic effects depending on its expression level. A low level of UBB^+1^ can stimulate a chaperone-buffering capacity, which likely masks the adverse effects of UBB^+1^ expression while simultaneously facilitating more robust prevention of protein aggregation. By contrast, UBB^+1^ accumulation inhibits proteasome processivity, which might foster increased aggregation and cytotoxicity of expanded polyQ proteins (Figure 2B) (de Pril et al., 2004; de Pril et al., 2010). Drugs that effectively inhibit or induce protein aggregate removal are still under development. Perhaps a targeted-clearance strategy based on AUTAC (autophagy-targeting chimera) could be designed to target UBB^+1^-labeled protein aggregates to induce their removal *via* autophagy (Takahashi et al., 2019). On the other hand, reduced protein turnover rates correlate positively with extended lifespan in several rodent species and long-lived animals (Pérez et al., 2009; Swovick et al., 2018), while an increase in overall protein degradation can be a hallmark of accelerated aging, as manifested in Hutchinson-Gilford Progeria Syndrome (Buchwalter and Hetzer, 2017). Could reducing proteasome activity *via* UBB^+1^ expression be a strategy used by aging or damaged cells to maintain their function for as long as possible? Future studies exploring the precise spatiotemporal expression patterns of UBB^+1^ could help to clarify this intriguing phenomenon and its effects on young and aging tissues.
